# Estrogen receptor beta as a novel target of androgen receptor action in breast cancer cell lines

**DOI:** 10.1186/bcr3619

**Published:** 2014-02-19

**Authors:** Pietro Rizza, Ines Barone, Domenico Zito, Francesca Giordano, Marilena Lanzino, Francesca De Amicis, Loredana Mauro, Diego Sisci, Stefania Catalano, Karin Dahlman Wright, Jan-ake Gustafsson, Sebastiano Andò

**Affiliations:** 1 Department of Pharmacy, Health and Nutritional Sciences, University of Calabria, Via P. Bucci, Arcavacata di Rende (CS), Cosenza 87036, Italy; 2 Department of Biosciences at Novum, Karolinska Institutet, S-14157 Huddinge, Sweden; 3 Department of Biology and Biochemistry, Center for Nuclear Receptors and Cell Signaling, University of Houston Director, Houston, Texas

## Abstract

**Introduction:**

The two isoforms of estrogen receptor (ER) alpha and beta play opposite roles in regulating proliferation and differentiation of breast cancers, with ER-alpha mediating mitogenic effects and ER-beta acting as a tumor suppressor. Emerging data have reported that androgen receptor (AR) activation inhibits ER-positive breast cancer progression mainly by antagonizing ER-alpha signaling. However, to date no studies have specifically evaluated a potential involvement of ER-beta in the inhibitory effects of androgens.

**Methods:**

ER-beta expression was examined in human breast cancer cell lines using real-time PCR, Western blotting and small interfering RNA (siRNA) assays. Mutagenesis studies, electromobility shift assay (EMSA) and chromatin immunoprecipitation (ChIP) analysis were performed to assess the effects of mibolerone/AR on ER-beta promoter activity and binding.

**Results:**

In this study, we demonstrate that mibolerone, a synthetic androgen ligand, up-regulates ER-beta mRNA and protein levels in ER-positive breast cancer cells. Transient transfection experiments, using a vector containing the human ER-beta promoter region, show that mibolerone increases basal ER-beta promoter activity. Site-directed mutagenesis and deletion analysis reveal that an androgen response element (ARE), TGTTCT motif located at positions −383 and −377, is critical for mibolerone-induced ER-beta up-regulation in breast cancer cells. This occurs through an increased recruitment of AR to the ARE site within the ER-beta promoter region, along with an enhanced occupancy of RNA polymerase II. Finally, silencing of ER-beta gene expression by RNA interference is able to partially reverse the effects of mibolerone on cell proliferation, p21 and cyclin D1 expression.

**Conclusions:**

Collectively, these data provide evidence for a novel mechanism by which activated AR, through an up-regulation of ER-beta gene expression, inhibits breast cancer cell growth.

## Introduction

Sex steroid hormones are critical for the development and progression of endocrine-dependent diseases, including breast cancers. Estrogen and androgen hormone signals are transduced via the action of specific members of a superfamily of nuclear steroid receptors that, functioning as ligand-activated transcription factors, are able to interact with a host of different coregulators to regulate gene transcription.

The roles of estrogen receptor (ER) alpha and beta in breast cancer pathogenesis are becoming increasingly elucidated by several clinical and *in vitro* studies. ER alpha mediates cancer-promoting effects of estrogen and has been shown to be an effective therapeutic target for decades [1]. In contrast, ER beta has a well known growth and invasion inhibitory activity in ERα-positive breast cancer cells, at least in part due to ER beta’s inhibition of ER alpha selective target gene expression, and can be considered as an endogenous partial dominant negative receptor [2,3]. Indeed, the progression of breast cancer is associated with a change in the expression ratio of the isoforms of ER, with ER alpha the predominant isoform expressed [4]. Moreover, compared with tumors expressing ER alpha alone, the co-expression of ER beta has been correlated with a more favorable prognosis [5] and decreased biological aggressiveness [6-9].

Androgen actions and androgen receptors (ARs) have been described in human breast cancers both *in vivo* and *in vitro*, but considerably less is known on their impact on this disease. Emerging evidence indicates that the androgen signaling pathway mainly exerts inhibitory effects on the growth of normal mammary epithelial cells and plays a protective role in the pathogenesis of breast cancer [10-13]. In particular, AR is present in approximately 70% to 90% of invasive breast carcinomas, a percentage equal to or higher than that of ER (70% to 80%) and its expression shows significant association with favorable clinicopathological characteristics, such as lower tumor grade, smaller tumor size, better outcomes and improved response to hormone therapy in ER-positive breast cancers [14-18]. *In vitro* studies have demonstrated that androgen signaling may counteract the proliferative effect of estrogens in AR-positive breast cancer cells [19], while over-expression of the AR markedly decreases ER alpha transcriptional activity in ER-positive breast cancer cells [20,21]. In these latter models, basal as well as estradiol-induced proliferation are inhibited by the non-aromatizable androgen 5-a-dihydrotestosterone (DHT) [21-23], through an increase in AR protein cell content [21], along with a block in G1 to S transition of the cell cycle [21,22]. AR-induced apoptosis has also been reported in these cell lines [24]. Recently, we have shown a direct down-regulation of the cyclin D1 gene expression by AR activation via interaction with the orphan nuclear receptor DAX1, as an additional mechanism involved in the androgen-dependent inhibition of ER-positive breast cancer cell growth [25].

Given the ability of AR to function as an anti-proliferative effecter by antagonizing ERα signaling [26], the aim of this study was to investigate whether ER beta may also be a target of androgen actions. Here, we demonstrate that mibolerone, a synthetic non-metabolizable androgen, up-regulates ER beta expression and gene promoter activity, through a direct binding of AR to a newly identified androgen response element (ARE) located in the human ER beta gene promoter.

## Methods

### Chemicals and reagents

Dulbecco’s Modified Eagle’s Medium/Nutrient Mixture F-12 Ham, DMEM, 100 bp DNA ladder, l-glutamine, penicillin, streptomycin, bovine serum albumin and phosphate-buffered saline were purchased from Invitrogen (Carlsbad, CA, USA), and Sephadex G50 spin columns and poly (dI-dC) from Roche (Indianapolis, IN, USA). GoTaq DNA polymerase, T4 polynucleotide kinase, dual luciferase kit, FuGENE 6 and CMV renilla luciferase plasmid were provided by Promega (Madison, WI, USA). The RETROscript kit and DNase I were purchased from Ambion (Austin, TX, USA). Aprotinin, leupeptin, phenylmethylsulfonyl fluoride, sodium orthovanadate, formaldehyde, NP-40, 3-(4,5-dimethylthiazol-2-yl)-2,5-diphenyl tetrazolium bromide (MTT), dimethyl sulfoxide, proteinase K, tRNA, IGF-1, DHT, bicatulamide(Cx) were from Sigma (Milan, Italy). Antibodies against cyclin D1, p21, GAPDH, and polymerase II (N20) were provided by Santa Cruz Biotechnology (Santa Cruz, CA, USA). Antibody anti-ER beta (clone 64–4, rabbit monoclonal immunoglobulin G (IgG), 1:1000 dilution) was purchased from Millipore. The ECL System, ^3^H thymidine and [γ32P]ATP were purchased from PerkinElmer (Wellesley, MA, USA), salmon sperm DNA/protein A agarose was from UBI (Chicago, IL, USA), and triazol, SYBR Green Universal PCR Master Mix was from Biosystems (Forster City, CA, USA).

### Cell cultures

The three human breast cancer cell lines MCF-7, ZR-75 and MDA-MD 231 were acquired in 2010 from American Type Culture Collection (ATCC, Manassas, VA, USA) where they were authenticated, stored according to the supplier’s instructions, and used within a month after frozen aliquots were resuscitated. MCF-7 cells were cultured in DMEM-F12 containing 5% fetal bovine serum (FBS), 1% l-glutamine and 1 mg/ml penicillin–streptomycin. ZR-75 cells were maintained in DMEM supplemented with 10% FBS, 1% l-glutamine and 1 mg/ml penicillin–streptomycin. MDA-MB- 231 cells were cultured in DMEM containing 5% FBS, 1% l-glutamine and 1 mg/ml penicillin–streptomycin.

Every four months cells were authenticated by single tandem repeat analysis at our Sequencing Core; morphology, doubling times, estrogen sensitivity, and mycoplasma negativity were tested (MycoAlert™, Lonza, Walkersville, MD, USA).

### Plasmids

The plasmids containing the human ER beta promoter 0 N region or its deletions (A: p − 1568/+315; B: p − 1283/+315, D: p − 355/+315) were a gift from Prof. Karin Dahlman-Wright (Department of Biosciences and Nutrition, Karolinska Institute, Sweden). C plasmid (p-400/+315) and mutated on ARE site C plasmid (M ARE mut) were generated by PCR using the following primers: forward 5′- ATATACGCGTAATCAGACATCTGTTCTGAATGACACTTATGTGAG −3′ and reverse 5′- ATATCTCGAGCGAAGGGGCGCTTACC −3′; forward 5′- ATATACGCGTAATCAGACATCTACCCTGAATGACACTTATGTG-3′. The amplified DNA fragments were digested with Mlu I and Xho I and ligated into pGL3-basic vector. The sequences were confirmed by nucleotide sequence analysis.

### Western blot analysis

Cells were treated as indicated before lysis. Equal amounts of cell extracts were subjected to SDS-PAGE, as described previously [27,28]. Blots are representative of at least three independent experiments.

### Real-time RT-PCR

The gene expression of ER beta, cyclin D1, p21 and GAPDH was evaluated by real-time RT-PCR. Total RNA was reverse transcribed with RETROscript kit. Diluted (1:3) cDNA (5 μl) was analyzed in triplicate by real-time PCR in an iCycler iQ Detection System (Bio-Rad, Hercules, California, USA) using SYBR Green Universal PCR Master Mix, following the manufacturer’s recommendations. Each sample was normalized on its GAPDH mRNA content. Primers used for the amplification were: forward 5′-CCCTGCTGTGATGAATTACAG −3′ and reverse 5′- TCGGTTCCCACTAACCTTCC-3′ (ER beta); forward 5–GCATGACAGATTTCTACCACTCC-3′ and reverse 5′-AAGATGTAGAGCGGGCCTTT-3′ (p21); forward 5′-CTGGAGGTCTGCGAGGAA-3′ and reverse 5′-GGGGATGGTCTCCTTCATCT-3′ (CYCD1); forward 5′-CCCACTCCTCCACCTTTGAC-3′ and reverse 5′-TGTTGCTGTAGCCAAATTCGTT-3′ (GAPDH). The relative gene expression levels were calculated as described [29].

### Transient transfection assays

MCF-7 cells were transiently transfected using the FuGENE 6 reagent with ER beta gene promoter, different deleted segments, and M ARE mut and treated as indicated. Empty vectors were used to ensure that DNA concentrations were constant in each transfection. Luciferase activities were assayed using the Dual Luciferase assay system (Promega) [30,31].

### Electrophoretic mobility shift assay (EMSA)

Nuclear extracts were prepared from MCF-7 cells treated with vehicle or mibolerone 10 nM for 16 hours as previously described [32,33]. The DNA sequences used as probe or as cold competitor are the following (mutations are shown as lowercase letters): 5′- AGAATCAGACATCTGTTCTGA ATGACA −3′, 5′ T GTCATTCAGAACAGATGTCTGATTCT-3′ (ARE); 5′- AGA ATCAGACATCTaccCTGAATGACA-3′, 5′- TGTCATTCAGggtAGATGTCTGATTCT −3 (mutated ARE). Probe generation and the protein-binding reactions were carried out as described [34]. For experiments involving antibodies, the reaction mixture was incubated with AR or IgG antibodies at 4°C for 12 hours before addition of labeled probe.

### RNA interference (RNAi)

MCF-7 cells were transfected with RNA duplex of stealth RNA interference (RNAi)-targeted for human ER beta mRNA sequence 5′-CACUUCUGCGCUGUCUGCAGCGAUU −3′ (Life Science, Hercules, California, USA) or with a stealth RNAi-negative control (Life Science) to a final concentration of 100 nM using Lipofectamine 2000 as recommended by the manufacturer [35,36].

### Chromatin immunoprecipitation assays

Cells were treated with vehicle or mibolerone 10 nM and after 16 hours the DNA/protein complexes were extracted as previously described [37]. The precleared chromatin was immunoprecipitated with anti-AR and anti-poymerase II antibodies. A normal mouse serum IgG was used as negative control. For each sample and input DNA, 5 μl were used for PCR amplification with the following primers flanking ARE sequence present in the ER beta promoter region: 5′- GGTTTCACCACTGGCTCCTT-3′ (forward) and 5′-ACTGATACAGCCAGTCTGGG-3′ (reverse). Final results were calculated using the ΔΔ Ct method, using input Ct values instead of the GAPDH mRNA. The basal sample was used as calibrator.

### Cell proliferation assays

Cell proliferation was determined by using the MTT assay as previously described [38] Data are representative of three independent experiments, each performed in triplicate. For [^3^H]thymidine incorporation, a total of 1 × 10^5^ cells was seeded onto 12-well plates in complete medium and then treated as indicated. Control (−) cells were treated with the same amount of vehicle alone (dimethylsulfoxide (DMSO)) that never exceeded a concentration of 0.01% (v/v). [^3^H]Thymidine incorporation was evaluated after a 24-hour incubation period with 1 μCi of [^3^H]thymidine per well. Cells were washed once with 10% trichloroacetic acid, twice with 5% trichloroacetic acid, and lysed in 1 ml of 0.1 M NaOH at 37°C for 30 minutes. The total suspension was added to 10 ml of Optifluor fluid and was counted in a scintillation counter.

### Statistical analysis

Data were analyzed for statistical significance using a two-tailed Student’s t-test, performed by Graph Pad Prism 4. Standard deviations (S.D.) are shown.

## Results

### Mibolerone increases ER beta expression in breast cancer cells

**Figure 1:**
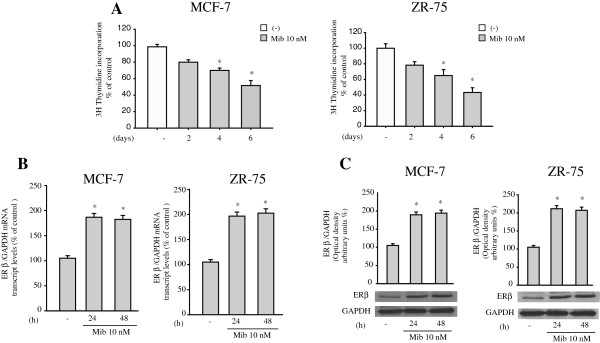
**Mibolerone up-regulates ER beta expression in breast cancer cells. (A) **^3^H Thymidine incorporation assays in MCF-7 and ZR-75 cells treated with vehicle (−) or mibolerone (Mib) 10 nM for two, four and six days. Columns, mean of three independent experiments, each performed with triplicate samples expressed as percent of control. **(B)** Total RNA was isolated from MCF-7 and ZR75 cells treated with vehicle (−) or Mib 10 nM for 24 and 48 hours, and reverse transcribed. cDNA was subjected to real time PCR using specific primers for ER beta and GAPDH. Each sample was normalized to its GAPDH mRNA content. Data represent the mean ± S.D. of values from three separate RNA samples expressed as a percentage of control assumed to be 100%. **(C)** Bottom panel, Western blot analysis of ER beta expression in total protein extracts from MCF-7 and ZR75 cells, treated with vehicle (−) or Mib 10 nM for 24 and 48 hours. GAPDH was used as loading control. Upper panel, the histograms represent the mean ± S.D. of three independent experiments in which band intensities were evaluated in terms of optical density arbitrary units and expressed as the percentage of the control assumed to be 100%. *, *P* <0.05 compared to vehicle-treated cells. ER, estrogen receptor.

We have previously demonstrated that the non-aromatizable androgen DHT decreased cell proliferation in a dose-dependent manner in ER-positive MCF-7 breast cancer cells [21,26]. In this study, to minimize the metabolic conversion of androgen to estrogenic compounds by cultured cells, we used the synthetic non-metabolizable androgen mibolerone to test its effects on cell proliferation by measuring changes in the rate of DNA synthesis (^3^H thymidine incorporation). We found similar inhibitory effects on the proliferation of ER-positive MCF-7 and ZR 75 breast cancer cells after two, four and six days of mibolerone treatment (Figure 1A). Given the known tumor repressive role of ER beta in breast cancer cell lines, we wondered whether AR also functions as an anti-proliferative effector in ER-positive breast cancer by affecting ER beta expression. First, MCF-7 and ZR75 breast cancer cells were treated with mibolerone for 24 and 48 hours and ER beta mRNA and protein levels were evaluated by real time RT-PCR and western blotting analysis. As shown in Figure 1B and C, mibolerone treatment increased ER beta expression at all times investigated in both MCF-7 and ZR75 cells.

**Figure 2:**
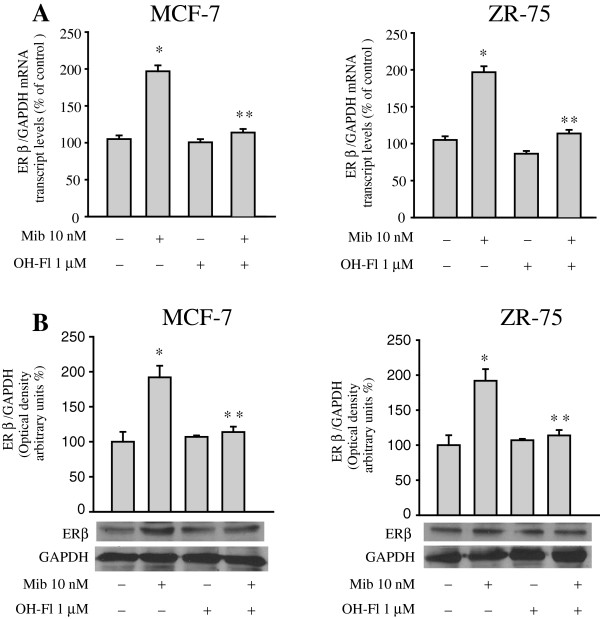
**Hydroxyflutamide (OH-Fl) reverses mibolerone’s effects on ER beta expression. (A)** Real-Time RT-PCR for analyzing ER beta mRNA levels in cells treated as indicated. Each sample was normalized to GAPDH RNA content. Data represent the mean ± S.D. of values from three separate RNA samples expressed as the percentage of control assumed to be 100%. **(B)** Bottom panel, Western blot analysis of ER beta expression in cells treated with vehicle (−) or Mib 10 nM in the presence or absence of OH-Fl 1 μM for 48 hours. GAPDH was used as loading control. Upper panel, the histograms represent the mean ± S.D. of three independent experiments in which band intensities were evaluated in terms of optical density arbitrary units and expressed as the percentage of the control assumed to be 100%. *, *P* <0.01 compared to vehicle treated-cells. **, *P* <0.01 compared to Mib treated cells. ER, estrogen receptor.

Clear evidence of the crucial role of AR in mediating these effects has been pointed out by the fact that the AR inhibitor hydroxyflutamide (OH-Fl) completely reversed the up-regulation of ER beta mRNA and protein content induced by mibolerone (Figure 2A and B). It is important to underline that using another androgen ligand, DHT, and an androgen antagonist, bicatulamide, we observed similar results on ER beta expression (Additional file 1: Figure S1B and S1C). Accordingly, bicalutamide reversed the inhibition mediated by both mibolerone and DHT on MCF-7 and ZR-75 cell growth (Additional file 1: Figure S1A).

### Activated AR up-regulates ER beta via an ARE site of its promoter

**Figure 3:**
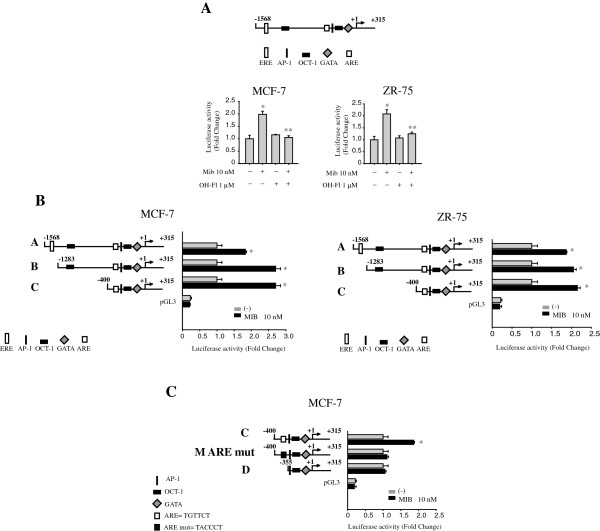
**ER beta promoter activity is up-regulated by mibolerone through an ARE site. (A)** Upper panel, schematic representation of construct of the ER beta gene promoter used in this study. Bottom panel, plasmid containing ER beta promoter fragment was transiently transfected in MCF-7 and ZR-75 cells treated with vehicle (−) or Mib 10 nM in the presence or absence of 1 μM OH-Fl. *, *P* <0.05 compared to vehicle-treated cells; *, *P* <0.05 compared to Mib-treated cells. **(B, C)** Left panel, schematic representation of constructs of the ER beta gene promoter used in this study. Right panel, plasmids containing ER beta promoter fragments were transiently transfected in cells treated with vehicle (−) or Mib 10 nM. After 24 hours of transfection, luciferase activities were normalized to the Renilla Luciferase as the internal transfection control and data were reported as fold change. The values represent the means ± S.D. of three different experiments each performed in triplicate. pGL3: basal activity measured in cells transfected with pGL3 basal vector. *, *P* <0.05 compared to vehicle-treated cells; *, *P* <0.05 compared to Mib-treated cells. ARE, androgen response element; ER, estrogen receptor.

To analyze if mibolerone might positively modulate ER beta gene transcription, MCF-7 and ZR75 breast cancer cell lines were transiently transfected with a luciferase reporter plasmid containing the human ER beta promoter region spanning from −1568 bp to +315 bp. As shown in Figure 3A, a significant increase in ER beta promoter activity was observed in cells treated with mibolerone, while this induction was abrogated in the presence of the AR inhibitor OH-Fl (Figure 3A). The human ER beta promoter contains multiple consensus sites for several transcription factors, including an AP-1 box, OCT-1, GATA, and ARE [39]. To identify the regions within the ER beta promoter responsible for mibolerone-mediated stimulatory effects, we transiently transfected both MCF-7 and ZR-75 cell lines with plasmids containing a series of 5′deleted segments of the human ER beta promoter. Schematic representation of these constructs is shown in Figure 3B. In transfection experiments performed using p-1568/+315 (A), p1256−/+315 (B) and p-400/+315 (C) plasmids, the responsiveness to mibolerone was still maintained (Figure 3B), suggesting that the region between −400 to +315 might be involved in the transactivation mechanisms exerted by mibolerone. Thus, we focused our attention on the latter construct, p-400/+315 (C), and we identified, upstream to the initiation transcription site, one ARE site, which is the putative effector of AR signaling. We observed that in MCF-7 cells transiently transfected with the ER beta promoter plasmid bearing the ARE-mutated site or with a deleted construct of ER beta promoter without the ARE site (p-355/+315, D), mibolerone was no longer able to induce ER beta promoter activity (Figure 3C). Similar results were obtained in ZR75 breast cancer cells (data not shown). Taken together, our findings demonstrated that the up-regulatory effects exerted by mibolerone on ERβ promoter gene expression require an ARE sequence motif.

### The AR protein is recruited at an ARE site to ER beta promoter region

**Figure 4:**
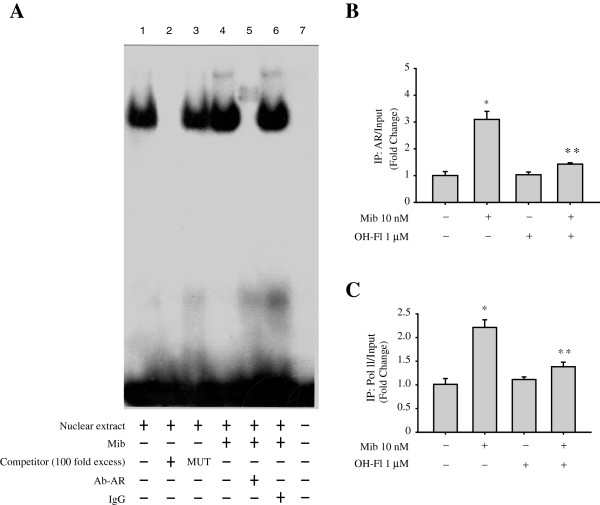
**Mibolerone recruits AR protein to an ARE site in the ER beta gene promoter. (A)** Nuclear extracts from MCF-7 cells treated with vehicle (−) or Mib 10 nM for 16 hours were incubated with a double-stranded ARE specific sequence probe labeled with [γ^32^P] ATP and subjected to electrophoresis in a 6% polyacrylamide gel (lanes 1 and 4). Competition experiments were performed adding as the competitor a 100-fold molar excess of unlabeled probe (lane 2 ) or a 100-fold molar excess of unlabeled oligonucleotide containing a mutated ARE motif (lane 3). Nuclear extracts from MCF-7 cells treated with Mib 10 nM for 16 hours were incubated with anti-AR (lane 5) or IgG (lane 6) antibodies, in the presence of the probe. Lane 7, probe alone. **(B,C)***,* MCF-7 cells treated with vehicle (−) or Mib 10 nM for 16 hours were cross-linked with formaldehyde and lysed. The pre-cleared chromatin was immune-precipitated with specific anti-AR, anti-polymerase II antibodies, and with a normal mouse serum (IgG) as a negative control. For each sample and input, a 5 μl volume was analyzed by real time PCR with specific primers, as detailed in the Methods Section, to amplify the ER beta promoter sequence containing the ARE site. The histograms represent the mean ± S.D. of three independent experiments. *, *P* <0.05 compared to vehicle-treated cells; *, *P* <0.01 compared to Mib-treated cells. AR, androgen receptor; ARE, androgen response element; ER, estrogen receptor; IgG, immunoglobulin G.

The specific role of the ARE motif in mediating the stimulatory role of mibolerone on ER beta gene expression was then investigated using EMSAs and chromatin immunoprecipitation (ChIP) assays. Using synthetic radiolabeled oligonucleotides bearing the ARE site present in the ER beta promoter region (Figure 4A, lane 1), we observed the formation of a protein complex in nuclear extracts from MCF-7 cells, which was abrogated by incubation with 100-fold molar excess of unlabeled probe (Figure 4A, lane 2), demonstrating the specificity of the DNA-binding complex. This inhibition was no longer observed when a mutated oligodeoxyribonucleotide probe was used as a competitor (Figure 4A, lane 3). Interestingly, treatment with mibolerone strongly increased the DNA-binding protein complex compared with control samples (Figure 4A, lane 4). The inclusion of the anti-AR antibody in the reaction supershifted the specific band, confirming the presence of this protein in the complex (Figure 4A, lanes 5). Non specific IgG did not affect AR complex formation (Figure 4A, lane 6).

Moreover, to better evaluate the functional importance of the ARE site at the ER beta promoter level, ChIP assays were performed. Protein-chromatin complexes were immunoprecipitated from MCF-7 cells treated with mibolerone using specific antibodies against AR or RNA-polymerase II (Figure 4B and C). Real-time PCR using primers spanning the AR binding element in the ER beta promoter region clearly showed an enhanced recruitment of AR upon treatment with mibolerone. These results were concomitant with an increased association of RNA-polymerase II to the ER beta regulatory region (Figure 4C), indicating that the chromatin in this region is probably in a better permissive environment for gene transcription.

### AR activation enhances ER beta expression in MDA-MB-231 breast cancer cells

**Figure 5:**
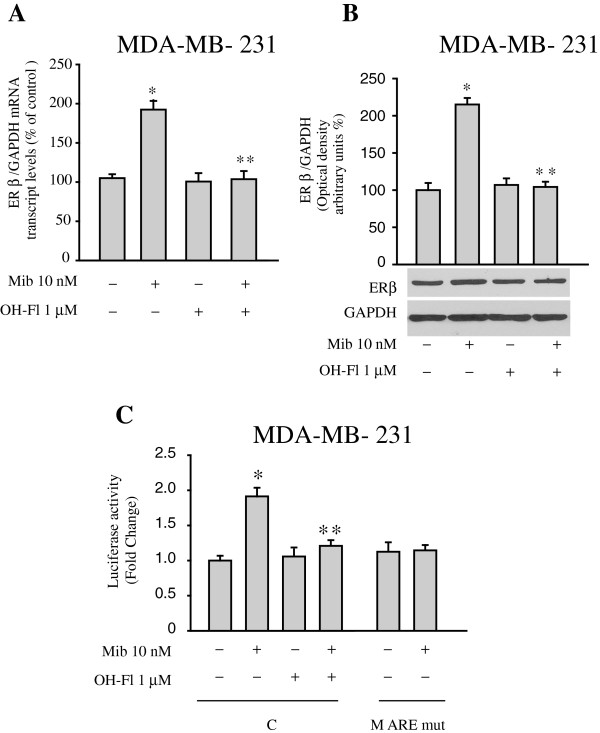
**Mibolerone increases ER beta expression in MDA-MB-231 breast cancer cells. (A)** Real-time PCR for ER beta mRNA expression in cells treated with vehicle (−) or mibolerone (Mib) 10 nM in the presence or absence of OH-Fl for 48 hours. Each sample was normalized for its GAPDH mRNA content. Data represent the mean ± S.D. of values from three separate RNA samples expressed as the percentage of the control assumed to be 100%. **(B)** Bottom panel, Western blot analysis of ER beta in total protein extracts from cells treated with vehicle (−) or Mib 10 nM in the presence or absence of OH-Fl for 48 hours. GAPDH was used as loading control. Upper panel, the histograms represent the mean ± S.D. of three independent experiments in which band intensities were evaluated in terms of optical density arbitrary units and expressed as the percentage of the control assumed to be 100%. **(C)** ER beta luciferase promoter activity in cells transfected and treated as indicated. The values represent the means ± S.D. of three different experiments each performed in triplicate. *, *P* <0.05 compared to vehicle-treated cells. **, *P* <0.05 compared to Mib-treated cells. ER, estrogen receptor.

To extend the results obtained, we also evaluated the effects of mibolerone on ER beta expression in the ER alpha-negative, ER beta-positive MDA-MB-231 breast cancer cell line. As previously shown for MCF-7 and ZR-75 cells, treatment with mibolerone for 48 hours enhanced ER beta mRNA and protein levels that were completely reversed in the presence of the androgen antagonist OH-Fl (Figure 5A and B). Again, treatment with mibolerone significantly increased ER beta promoter activity and this induction was abrogated both in cells treated with OH-Fl and in cells transfected with the ER beta promoter plasmid bearing the ARE mutated site (M ARE mut) (Figure 5C). Therefore, AR activation resulted in an up-regulation of ER beta expression and activity in different cellular backgrounds.

### ER beta knockdown reverses mibolerone’s effects on cell proliferation

Since the anti-proliferative effects of ER beta have been associated with a repression of cyclin D1 expression and activation of growth-inhibitory genes, such as p21 (WAF1) [40], and these genes are important in mediating AR inhibitory signaling [41], we aimed to examine whether ER beta may be involved in modulation of the expression of p21 and cyclin D1 induced by mibolerone.

**Figure 6:**
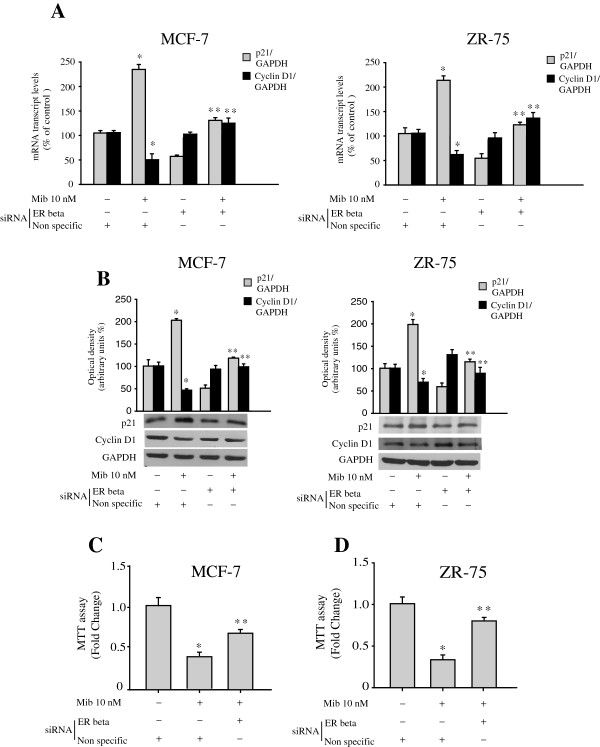
**ER beta silencing counteracts the effects of mibolerone on p21, cyclin D1 expression and cell proliferation. (A)** RNA was extracted from MCF-7 cells treated with vehicle (−) or Mib 10 nM for 48 hours in the presence of a non-specific or an ER beta siRNA, reverse transcribed and cDNA was subjected to Real-Time RT-PCR for p21 and cyclin D1 mRNA expression. Each sample was normalized to GAPDH mRNA content. Data represent the mean ± S.D. of values from three separate RNA samples expressed as the percentage of the control (−) assumed to be 100%. **(B)** Western blot analysis for p21 and cyclin D1 expression in cells treated with vehicle (−) or Mib for 48 hours in the presence of a non-specific or an ER beta siRNA. GAPDH was used as the loading control. The histograms represent the mean ± S.D. of three independent experiments in which band intensities were evaluated in terms of optical density arbitrary units and expressed as the percentage of the control assumed to be 100%. **(C, D)** MTT assays in MCF-7 and ZR-75 cells transfected and treated as indicated. Results are expressed as fold change ± S.D and are representative of three different experiments each performed in triplicate. *, *P* <0.01 compared to vehicle-treated cells. **, *P* <0.01 ER beta siRNA-transfected cells compared to Mib-treated cells. ER, estrogen receptor; MTT, 3-(4,5-dimethylthiazol-2-yl)-2,5-diphenyl tetrazolium bromide.

To this aim, ER beta siRNA knockdown experiments were performed in both MCF-7 and ZR-75 breast cancer cells treated with mibolerone. As expected, mibolerone treatment induced an increase of p21 expression along with a reduction of cyclin D1 expression. Silencing of ER beta gene expression (evaluated by Western blot, Additional file 2: Figure S2) counteracted at least in part the effects of mibolerone on p21 and cyclin D1 expression at both the mRNA and protein levels. No changes were observed after transfection of cells with a scrambled siRNA control (Figure 6A and B). Accordingly, ER beta gene silencing partially reversed the inhibition mediated by mibolerone on MCF-7 and ZR-75 cell growth (Figure 6C and E), suggesting how the anti-proliferative effects exerted by mibolerone may also be related to an induction of ER beta levels.

## Discussion

**Figure 7:**
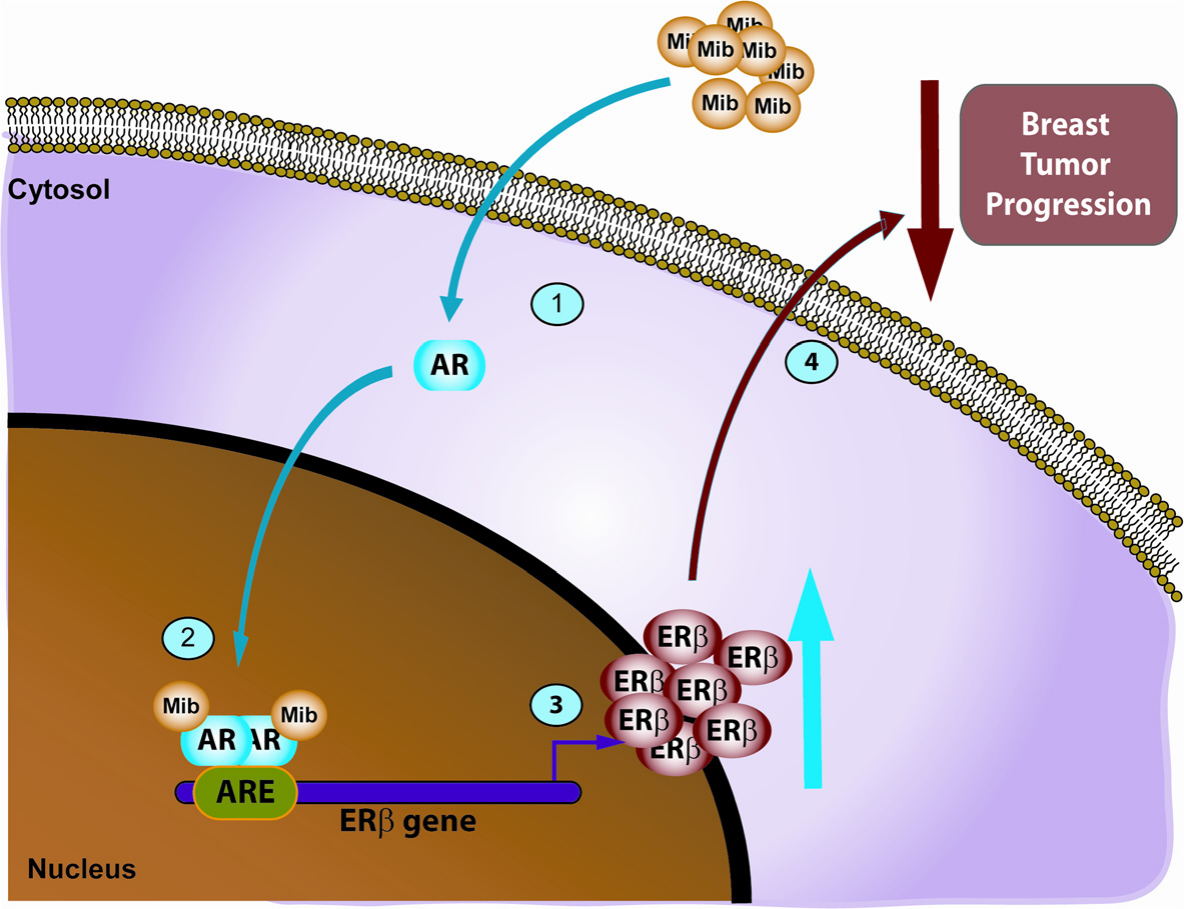
**Proposed working model of the AR-mediated regulation of ER beta expression in breast cancer cell lines.** In MCF-7 and ZR-75 cells, upon mibolerone treatment, AR is recruited on the ARE-containing region of ER beta promoter, leading to an increase in ER beta expression and resulting in an inhibition of tumor growth progression. AR, androgen receptor; ARE, androgen response element; ER, estrogen receptor.

In this study, we show, for the first time, that androgens increase ER beta gene expression in ERα-positive breast cancer cells. This occurs through an enhanced recruitment of AR to the ARE site located at −383 to −377 bp up-stream of the initiating transcription site within the human ER beta promoter region (Figure 7).

The ER expression is a well established marker in clinical practice, reflecting the biology of the tumor, the prognosis and the prediction of responsiveness to endocrine therapy [42,43]. However, the clinical significance of other endocrine-related pathways such as androgen and AR signaling has been recently proposed in primary breast cancer patients [44]. Indeed, several studies have suggested that AR expression correlates with favorable clinicopathological characteristics at diagnosis, including lower histologic grade, smaller tumor size, negative nodal status and ER positivity, and better outcomes among study populations as a whole and the subgroups who received endocrine therapy alone [45,46]. Despite the growing evidence highlighting the potential prognostic and/or predictive role of AR in breast cancer, the mechanisms by which androgen signaling may affect tumor progression still remain not well clarified.

Several *in vitro* studies suggest that androgens may exert a divergent role in breast cancer cells [47]. For instance, it has been demonstrated that AR cooperates with ERs at the non-transcriptional level leading to Src activation and stimulation of DNA synthesis [48-50]. Nevertheless, most *in vitro* and *in vivo* studies indicate that activated AR exerts an anti-estrogenic, growth-inhibitory influence in ERα-positive luminal breast cancers, partly dependent on its ability to antagonize ERα signaling through several mechanisms [51,52].

However, no specific studies have investigated the potential crosstalk between AR and ER beta. Here, we have demonstrated that treatment with the synthetic non-metabolizable androgen mibolerone induced an increase of ER beta expression both in terms of mRNA levels and protein content in MCF-7 and ZR-75 breast cancer cells. These effects are completely reversed by the AR inhibitor OH-flutamide, confirming the role of AR in the up-regulation of ER beta mediated by mibolerone. We also reproduced similar results in ER alpha negative, ER beta positive breast cancer cells MDA-MB-231 suggesting that the action of androgens on ER beta expression may represent a general mechanism not related to cell specificity.

In humans, different isoforms of the ER beta mRNA which diverge in their 5′-untranslated regions were identified [53]. They were generated by alternative splicing of two upstream exons, exon 0 K and exon 0 N, to exon 1, indicating that transcription of the human ER beta gene occurs from at least two different promoters. The ER beta gene promoter 0 N has been cloned and characterized [39]. Sequence analysis of the 5′-flanking region of ER beta promoter 0 N has shown the presence of several consensus transcriptional factor binding sites and cis regulatory elements [39], including an AP-1 box, OCT-1, GATA, and ARE. AREs are defined as chromosomal regions to which the AR is recruited in order to modulate gene expression in an androgen-dependent manner [54] Although the sequence 5′-AGAACAnnnTGTTGT-3′ has been described as the canonical ARE presenting an inverted repeat [55], different studies revealed that AREs can significantly differ from this ‘classical’ sequence [56]. For instance, AREs can be arranged as direct repeats and differences in the sequence and arrangement of AR-binding sites have been described which seem to mediate variable affinity and specificity of AR [57,58]. Indeed, ChIP-on-chip data revealed that the AR binds to genomic regions that contain DNA elements which might consist of simple 5′-TGTTCT-3′-like monomer binding sites [54,59,60]. Our functional experiments using ER beta promoter-deleted or mutated constructs have shown that the ARE sequence is an important prerequisite for the up-regulatory effects of mibolerone on ER beta promoter activity. These results were well supported by electrophoretic mobility shift assays, which revealed a marked increase in a specific DNA-binding complex in nuclear extracts from MCF-7 cells treated with mibolerone. This complex was immune-supershifted by an anti-AR antibody, indicating the presence of AR in the complex. Furthermore, ChIP analysis clearly showed an enhanced recruitment of AR to the ARE site within ER beta gene promoter, that was concomitant with an increase in RNA polymerase II occupancy, supporting the positive role for mibolerone in inducing ER beta gene transcriptional machinery.

ER beta has been shown to inhibit human breast cancer cell proliferation by repressing transcription of the c-myc, cyclin D1 and cyclin A genes and increasing the expression of the cyclin-dependent kinase inhibitors p21^Waf1/Cip1^ and p27^Kip1^, leading to cell cycle arrest in the G2 phase [5,40,61]. Moreover, p21 up-regulation and cyclin D1 down-regulation have been identified as important events able to mediate AR signaling [25,41]. In accordance with these findings, we demonstrate that silencing of ER beta gene expression reduced both protein and mRNA expression of p21 induced by mibolerone, while it increased both protein and mRNA expression of cyclin D1 reduced by mibolerone. In addition, the anti-proliferative effects exerted by androgens were partially reversed in the presence of ER beta siRNA knockdown in both MCF-7 and ZR-75 breast cancer cells, suggesting how the growth inhibitory effects exerted by mibolerone may also be related to an induction of ER beta levels.

## Conclusions

We suggest that induction of ER beta expression by mibolerone may play a critical regulatory role in ER-positive cells, addressing prospectively that combined agents able to potentiate ER beta and AR signalings may be useful to inhibit breast cancer cell growth and progression.

## Abbreviations

AR: androgen receptor; ARE: androgen response element; bp: base pair; ChIP: chromatin immunoprecipitation; DHT: 5-a-dihydrotestosterone; EMSA: electrophoretic mobility shift assay; ER: estrogen receptor; IgG: immunoglobulin G; OH-Fl: hydroxyflutamide; MTT: 3-(4,5-dimethylthiazol-2-yl)-2,5-diphenyl tetrazolium bromide; RT-PCR: reverse transcriptase-polymerase chain reaction; siRNA: small interfering RNA.

## Competing interests

The authors declare that they have no competing interests.

## Authors’ contributions

PR and IB are co-first authors of this work. PR and SA designed all the experiments. DZ performed siRNA experiments, generated the deleted and mutated constructs and tested them in functional assays. FG performed western blotting and real-time RT-PCR. ML carried out ChIP assays. FDA performed EMSA experiments. LM carried out cell proliferation assays. The paper was written by PR, IB and SA. DS and SC prepared figures and assisted in editing the manuscript. KDW and JAG were involved in manuscript revision. All authors were involved in drafting the manuscript or revising it critically for important intellectual content. All authors read and approved the final manuscript.

## Supplementary Material

Additional file 1: Figure S1 Hydroxyflutamide (OH-Fl) and Bicatulamide (Cx) reverse mibolerone’s or DHT's effects on cell proliferation and ER beta expression in breast cancer cells. (A) MCF-7 and ZR-75 cells were treated for six days with the indicated concentrations of Mib, DHT, OH-Fl and Cx. MCF-7 and ZR-75 cell proliferation were evaluated by [3H]thymidine incorporation analysis. Columns, mean of three independent experiments each performed with triplicate samples expressed as percent of control. (B) Bottom panel, Western Blot analysis of ER beta expression in cells treated for 48 hours as indicated. GAPDH was used as loading control. Upper panel, the histograms represent the mean ± S.D. of three independent experiments in which band intensities were evaluated in terms of optical density arbitrary units and expressed as the percentage of the control assumed to be 100%. (C) Real-Time RT-PCR for analyzing ER beta mRNA levels in cells treated as indicated. Each sample was normalized to GAPDH RNA content. Data represent the mean ± S.D. of values from three separate RNA samples expressed as percentage of control assumed to be 100%. *, *P* <0.01 compared to vehicle treated-cells. Click here for file

Additional file 2: Figure S2 Knockdown of ER beta in MCF-7 cells. Western blot analysis for ER beta in MCF-7 cells transfected with non-specific siRNA (−) or targeted against human ER beta (100 nM) for 48 hours. GAPDH was used as a loading control. LNCaP (+) was used for positive control. Click here for file
