# Supplementary material for: Estrogen receptor beta as a novel target of androgen receptor action in breast cancer cell lines
Source: Breast Cancer Res. 2014 Feb 19;16(1):R21. doi: 10.1186/bcr3619 (PMC3978907; doi:10.1186/bcr3619)
Supplement: Additional file 1: Figure S1 — Hydroxyflutamide (OH-Fl) and Bicatulamide (Cx) reverse mibolerone’s or DHT's effects on cell proliferation and ER beta expression in breast cancer cells. (A) MCF-7 and ZR-75 cells were treated for six days with the indicated concentrations of Mib, DHT, OH-Fl and Cx. MCF-7 and ZR-75 cell proliferation were evaluated by [3H]thymidine incorporation analysis. Columns, mean of three independent experiments each performed with triplicate samples expressed as percent of control. (B) Bottom panel, Western Blot analysis of ER beta expression in cells treated for 48 hours as indicated. GAPDH was used as loading control. Upper panel, the histograms represent the mean ± S.D. of three independent experiments in which band intensities were evaluated in terms of optical density arbitrary units and expressed as the percentage of the control assumed to be 100%. (C) Real-Time RT-PCR for analyzing ER beta mRNA levels in cells treated as indicated. Each sample was normalized to GAPDH RNA content. Data represent the mean ± S.D. of values from three separate RNA samples expressed as percentage of control assumed to be 100%. *, P <0.01 compared to vehicle treated-cells. [file bcr3619-S1.pdf]

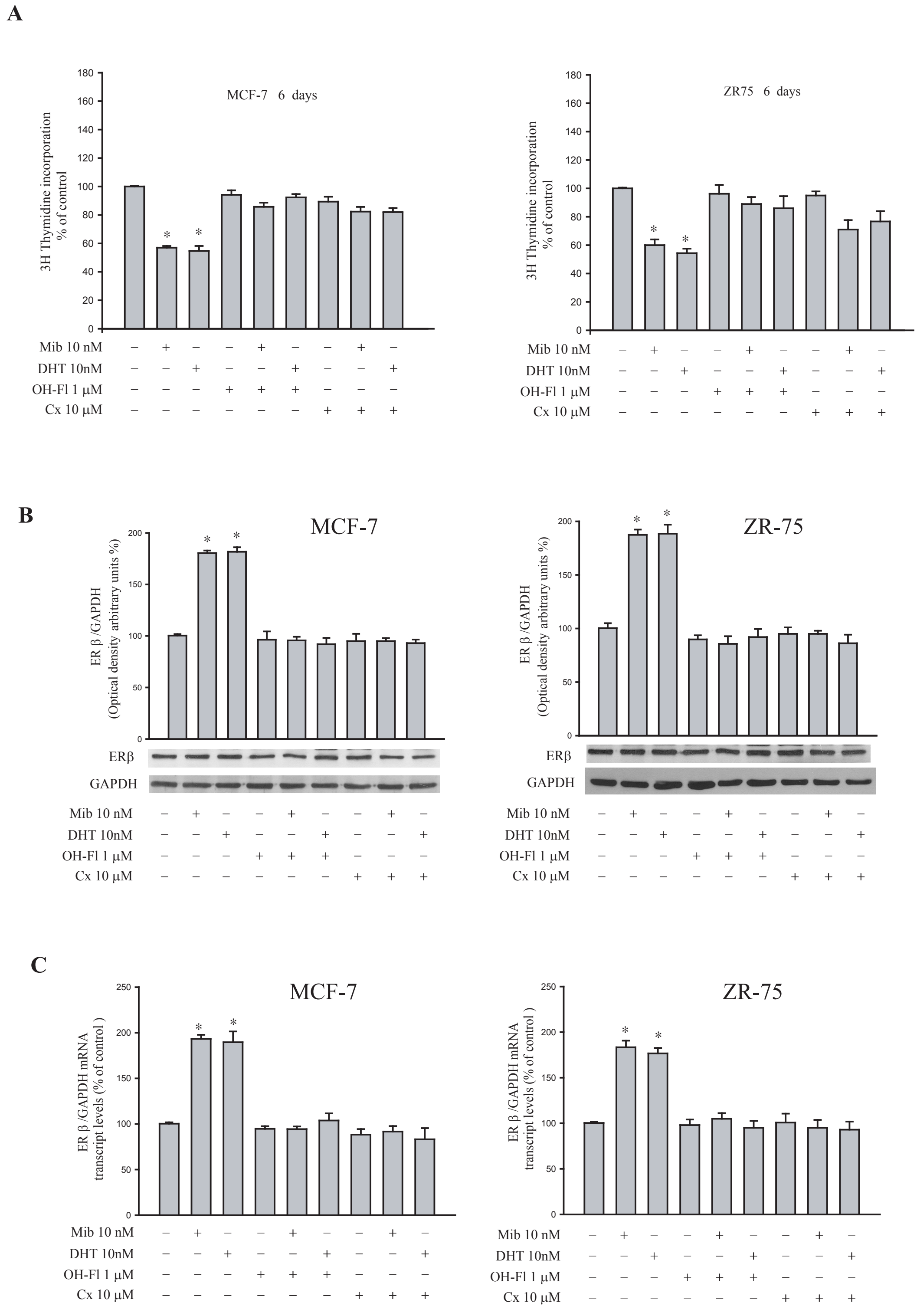


**Additional file 1, Figure S1. Hydroxyflutamide (OH-Fl) and Bicatulamide (Cx) reverse mibolerone's or DHT's effects on cell proliferation and ER beta expression in breast cancer cells.**

(**A**) MCF-7 and ZR-75 cells were treated for 6 days with the indicated concentrations of Mib, DHT, OH-Fl and Cx. MCF-7 and ZR-75 cell proliferation were evaluated by [3H]thymidine incorporation analysis. Columns, mean of three independent experiments each performed with triplicate samples expressed as percent of control;
(**B**) Bottom panel, Western Blot analysis of ER beta expression in cells treated for 48 hours as indicated. GAPDH was used as loading control. Upper panel, the histograms represent the mean ± S.D. of three independent experiments in which band intensities were evaluated in terms of optical density arbitrary units and expressed as the percentage of the control assumed to be 100%. (**C**) Real-Time RT-PCR for analyzing ER beta mRNA levels in cells treated as indicated. Each sample was normalized to GAPDH RNA content. Data represent the- mean ± S.D. of values from three separate RNA samples expressed as percentage of control assumed to be 100%.
*, P<0.01 compared to vehicle treated-cells.
