# Supplementary material for: Estrogen receptor beta as a novel target of androgen receptor action in breast cancer cell lines
Source: Breast Cancer Res. 2014 Feb 19;16(1):R21. doi: 10.1186/bcr3619 (PMC3978907; doi:10.1186/bcr3619)
Supplement: Additional file 2: Figure S2 — Knockdown of ER beta in MCF-7 cells. Western blot analysis for ER beta in MCF-7 cells transfected with non-specific siRNA (−) or targeted against human ER beta (100 nM) for 48 hours. GAPDH was used as a loading control. LNCaP (+) was used for positive control. [file bcr3619-S2.pdf]

MCF-7

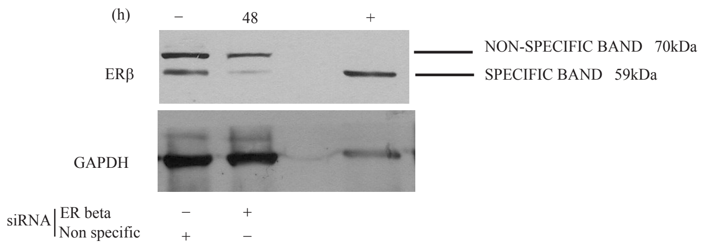

Additional file 2, Figure S2. Knockdown of ER beta in MCF-7 cells.
Western blot analysis for ER beta in MCF-7 cells transfected with non-specific siRNA (-) or targeted against human ER beta (100 nM) for 48h. GAPDH was used as a loading control. LNCaP (+) was used for positive control.
